# Public involvement in health research: what does ‘good’ look like in practice?

**DOI:** 10.1186/s40900-020-0183-x

**Published:** 2020-03-31

**Authors:** Kristin Liabo, Kate Boddy, Silvia Bortoli, Jenny Irvine, Heather Boult, Mary Fredlund, Neil Joseph, Gretchen Bjornstad, Christopher Morris

**Affiliations:** 1 NIHR Applied Research Collaboration South West Peninsula, University of Exeter Medical School, St Luke’s Campus, Exeter, EX1 2LU UK; 2 Living Systems Institute, University of Exeter College of Life and Environmental Sciences, Stocker Road, Exeter, EX4 4QD UK; 3 Peninsula Childhood Disability Research Unit (PenCRU), University of Exeter Medical School, St Luke’s Campus, Exeter, EX1 2LU UK; 4 NIHR Collaboration for Leadership in Applied Health Research and Care (CLAHRC) North West Coast, Lancaster University, Lancaster, UK; 5 Peninsula Public Involvement Group, NIHR Applied Research Collaboration South West Peninsula, University of Exeter Medical School, St Luke’s Campus, Exeter, EX1 2LU UK; 6 PenCRU Family Faculty, Peninsula Childhood Disability Research Unit, University of Exeter Medical School, St Luke’s Campus, Exeter, EX1 2LU UK; 7 Public Adviser Forum, NIHR Applied Research Collaboration North West Coast, Lancaster University, Lancaster, LA1 4YX UK

**Keywords:** Patient and public involvement, Evaluation, Literature review, Co-production

## Abstract

**Plain English summary:**

**Background**

Patient and public involvement means researchers working with members of the public, patients or carers to jointly plan and carry out research.

**Aim**

This article is written by members of three involvement groups, and the university employees that they work with. We wanted to jointly reflect on what enables our collaborative work, and what the challenges are for everyone involved.

**What we did and how we did it**

We wanted to establish what the literature defines as ‘good’ public involvement and compare this with processes and practices in our involvement groups. We therefore carried out a literature review and each group met separately to discuss what characterises good involvement, and what the challenges are. From these discussions we developed a set of descriptions about each group. We compared the literature review findings with what came out of the discussions within the involvement groups.

**Findings**

Some of the involvement principles from the literature were similar to the priorities of the involvement groups. In addition, the groups identified characteristics of ‘good’ involvement practice that were not reported in the literature: passion and enthusiasm, informal and welcoming meeting spaces, and opportunities to share lived experiences. In this article we present examples of how principles for good involvement are practiced in these groups, and difficulties we have experienced.

**Abstract:**

**Background**

Patient and public involvement is important for producing relevant and accessible health research. Evidence of impact from involvement is growing, but there is also a need for research on how to create conditions for meaningful collaborations between researchers and public advisers.

**Objective**

We report on a co-produced self-reflective evaluation of involvement practices in three UK research programmes.

**Methods**

A structured review identified research-based principles for ‘good’ public involvement in research. In parallel, members of three involvement groups co-developed statements on how the groups work, and enablers and challenges to collaborative research. The author team analysed these statements using the findings from the review.

**Results**

We identified 11 international articles reporting research-based principles for involvement published between 2013 and 2017. We identified five ‘values’ and seven ‘practice principles’ for ‘good’ involvement. There was convergence between these principles and the priorities of the involvement groups. But the groups also identified additional good involvement practice that were not reported by the literature: passion, enthusiasm, informal and welcoming meeting spaces, and opportunities to share lived experiences. We present examples of how principles for good involvement are practiced in these groups, and highlight principles that have been challenging to implement.

**Conclusions**

Ongoing appraisal of public involvement is crucial. We present a process for self-evaluation, illuminate what ‘good’ means to researchers and public advisers involved in research, and identify areas for improvement. We conclude that provision of resources that enable support to public advisers in turn enable universities and research teams to implement other principles of good involvement.

## Background

Collaborative research between patients, carers, members of the public, communities and researchers is now mandated by funding agencies in many countries [1–5]. With the formal push for researchers to initiate such collaborations has come a desire to establish what ‘good’ public involvement in health research looks like.

A recent systematic review mapped four essential *components* of involvement in research [6]. Standards for involvement are also developed by organisations supporting it [7, 8]. Published principles or components of involvement may tell us what we should aspire to, but often lack practical details on implementing public involvement in practice [9]; nor do they illustrate to public advisors what they should expect of researchers, practically [7, 8]. The details of involvement matter, and exploring the details can help us avoid tokenistic involvement [10].

In 2015 three involvement groups, researchers and involvement facilitators participated in a two-day learning exchange event. After this event we collaborated on a project to jointly examine our practices for enabling public involvement. This article describes our way of self-assessing against involvement standards, sharing experiences of what works in involvement, and identifying how to improve.

The research questions we set out to address in our evaluation were:

- Research question 1: What are the commonly agreed principles for good public involvement in research? Our self-evaluation started before publication of the standards for involvement led by the UK National Institute for Health Research [8]. Due to a lack of established standards at the time, we carried out a literature review. Nevertheless, our findings speak to these standards and provide examples for how they can be implemented.
- Research question 2: How do these principles for good involvement, identified in the literature, compare with the views of members of our three involvement groups? This question was the main focus for our workshops with each group.
- Research question 3: What do these involvement principles look like in practice? This was the primary aim of our work, to identify how involvement standards can best be implemented and practiced.

This article is co-written by members of the public, patients and parent carers, public involvement facilitators and researchers. It conveys multiple perspectives from our collaborative work across the three involvement groups on how we ‘live’ principles for good involvement. Three of the authors are members of each respective group (HB, MF, NJ). The other authors work with one of the groups in a professional capacity as researchers (GB, CM), involvement facilitators (SB, JI) or both (KB, KL). The author group met regularly over 2 years.

## Involvement contexts

The public advisors who co-authored this article are residents of specific neighbourhoods, members of community groups, patients, carers, and parents of children with long-term neurological conditions. They are all members of long-standing public involvement groups set up and run by university-based health researchers. A description of each group is provided in Table 1.

**Table 1:** Overview of the involvement groups

Peninsula Childhood Disability Research Unit (PenCRU) Family Faculty	PenCRU is a research unit focusing on childhood neurodisability. PenCRU is a partnership between researchers, families and health professionals. Staff includes researchers, a Family Involvement Coordinator and an administrator. Parent carers are members of the PenCRU Family Faculty, which currently has over 200 contacts. Parent carers are involved in all aspects of the research cycle. For substantive projects ‘working groups’ are convened whereby parents participate in meetings and/or input research via email and telephone. Membership of the Family Faculty does not commit members beyond receiving emails about PenCRU activities and opportunities to get involved.
Adviser Forum to the CLAHRC^a^ North West Coast (NWC)	There are more than 100 members of the Adviser Forum who are involved in a CLAHRC NWC study or implementation activity. Public advisers also hold positions on the CLAHRC NWC steering board, management group and the committee that approves funding for new studies. Each CLAHRC NWC research study consists of three partners; A University (Lancaster, Liverpool or Central Lancashire) partner, a partner from a local authority or the NHS, and community members. There is one full-time public engagement facilitator and a part-time assistant to support the group.
Peninsula Public Involvement Group (PenPIG) for CLAHRC South West Peninsula (PenCLAHRC)	PenPIG is a public advisory group, or ‘critical friend’, for PenCLAHRC, which is a partnership of local NHS Trusts across Somerset, Devon and Cornwall, plus the Universities of Exeter and Plymouth. The group has representation on PenCLAHRC’s Management Board, and members are involved in research prioritisation, research funding applications, on-going research and dissemination. Three researchers and one administrator support PenPIG members with their involvement in research. This team also helps researchers convene patient-specific groups beyond PenPIG.

^a^Collaboration for Leadership in Applied Health Research and Care, programme grants funded by the UK National Institute for Health Research. New name from 2019 is the NIHR Applied Research Collaborations (ARC)

## Evaluation design and methods

We wanted a set of standards to self-assess against, and therefore conducted a structured review of literature on involvement. Each group co-produced descriptions and statements about how they work. We assessed these statements against the findings from our literature review.

**Fig. 1:**
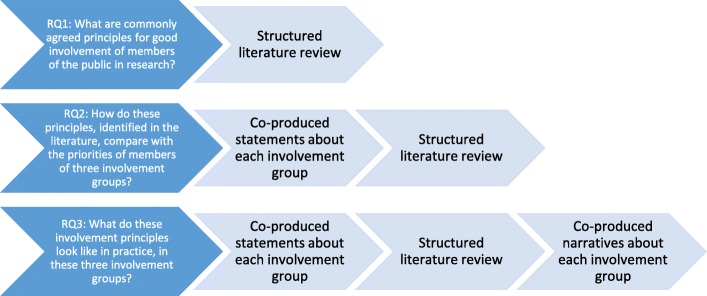
Self-reflective evaluation questions and design

The questions and design of our evaluation are shown in Fig. 1.

The co-production workshops with each group followed a format designed by JI. The aim of the workshop was to enable members to analyse the involvement structures and practices within their own group, and to generate text that reflected priorities and views of group members. A truncated version of the workshop plan is shown in Table 2.

**Table 2:** Overview of workshop content

Time	Question	Activity
15 min	**Introduction:** Introduce the topic using the lay summary. • Explain that three Involvement groups will each complete the same exercise separately to feed in to the work. • The exercise is based around three questions which are intended to prompt thought and discussion in the group: • There are no right or wrong answers to the questions, the exercise will help paint a picture of what makes each group unique. • **Question: How is this group best described in terms of what it does, and how it is set up?** Recap group function as a reminder to give ideas.	
5 min	How is this group best described in terms of what it does, and how it is set up?	**Work alone to answer the question** Members to think about the question and write individual answers/important areas/keywords on post it notes. One topic/idea per post-it note. (Prompt words: responsibilities, relationships, activities, roles and motivations)
10 min	How is this group best described in terms of what it does, and how it is set up?	**Work in pairs to answer the question** Members to turn to their neighbour and discuss the question, put further ideas on post it notes. After 5 min, one person per pair to feedback an area they have thought of.
15 min	How is this group best described in terms of what it does, and how it is set up?	**Work with the whole table to organise answers on the post-its into categories** • Tables can choose a member to act as facilitator to smooth the process of agreeing categories and to get the task done. • Duplicate answers on post-its stacked together to represent one post-it. At the end each table will have their words sorted in to categories.
10 min	Sum up the exercise by finding out what categories and areas the tables have chosen, asking for feedback from tables.	
10 min	**Coffee break**	Facilitators put fresh flipchart paper and post it notes on tables, collect other post it notes in for keeping.
30 min	What makes this group work?	Address this question first alone (5 min), then in pairs (10 min), then with the Table (15 min), as with the previous question.
5 min	Move the flipcharts with categories and post it notes onto one table/area where they can be easily seen and accessed for voting	
10 min	What makes this group work?	**Bean voting by individuals:** Each person has 12 dried beans, these are voting beans which **represent importance.** It is each individual’s decision where these are placed, not a group decision. Each person can place up to 3 beans on a post-it note or they can place a bean on 12 different post-it notes.
15 min	**Facilitators count bean votes and record outcomes – attendees can refresh drinks etc, Feedback to group**	
5 min	**Break**	
5 min	What are the challenges facing the group? or being a member?	Address this question first alone (5 min), then in pairs (10 min), then with the Table (15 min), as with the previous question.
5 min	**Move the flipcharts with categories and post it notes onto one table/area where they can be easily seen and accessed for voting**	
10 min	What are the challenges facing this group, or being a member?	**Bean voting by individuals,** as with previous voting
10 min	What are the challenges facing this group, or being a member?	Facilitators count bean votes and record outcomes, feedback to group
10 min	**Wrap up session**	What will happen next, Admin forms

The workshop was structured around three key questions: 1) How is this group best described? 2) What makes this group work? 3) What are the challenges facing this group? The groups prioritised answers to each question through debate and voting, and the prioritised responses were tabulated and compared across the three groups. Authors from each group wrote a narrative based on their group’s workshop discussions. These detailed the group’s structures and function, and gave depth to the information provided in the workshops. The narratives were circulated within groups for verification, then reviewed by authors from other groups.

## Structured literature review

For the structured review we defined *principles* as dimensions or factors that contribute to involvement being ‘good’. ‘Good’ involvement in this context is experienced as positive and meaningful by public advisors and researchers, and has influence on how research is designed and conducted. We only included reports that were based on research, such as stakeholder interviews or a Delphi process. The aim of the review was not to produce a stand-alone systematic review, but to have a robust framework to compare ourselves to.

Pragmatic decisions were taken to target the relevant literature using a combination of thesaurus headings and free-text terms. A database entry limit of January 1st 2013 was applied to capture literature published after the most recent systematic review we could find on the topic [6]. We searched the following databases (January 2013 to August 2017): Applied Social Science Index and Abstracts (ProQuest), Medline (Ovid), Scopus and web of Science. Forwards and backwards citation searching was conducted in web of Science and Google Scholar. Relevant non-indexed journals and bibliographies of included publications were hand-searched, along with online searches to locate national-level involvement standards.

We included articles that described a set of principles or standards, a model or a framework of categories developed from data on public involvement. We were not looking for descriptions of involvement facilitation tools, but rules or guidelines for ways of working with patients or members of the public. We double screened 1373 hits based on titles and abstracts, then double screened initial includes (21 full-text articles), and 11 met the inclusion criteria.

## Analysis

To address research question 1, KB and KL independently extracted principles from included articles and organised them into emerging themes. This was done by grouping principles that conveyed similar meaning and values. All authors revised the themes and made adjustments to the analysis.

To address research question 2, we compared group members’ prioritised statements about their involvement group with themes identified in the literature.

To address research question 3, we used the synthesised themes from the literature review to thematically code each group’s narratives. Two authors from each group independently coded the narratives of the other two groups. The coding was compared and discrepancies resolved by discussion. This enabled us to identify practical responses to principles for good involvement. We selected examples within each group to illustrate implementation of some of the principles. We also identified principles that were challenging to implement.

## Results

### Research question 1: what are the commonly agreed principles for good public involvement in research?

We included 11 international publications in our review [7, 11–20]. The synthesised principles for ‘good’ involvement fell within two overarching categories; ‘values’ and ‘practicalities’. By ‘value’ we mean an ideal of importance to public involvement. For example ‘purposeful’ is a value because it doesn’t describe an action but is an overarching ideal that people should aspire to achieve. By ‘practicality’ we mean something that enables the value. For example, ‘involved people kept updated’ is a practical way of making the involvement ‘purposeful’. The five value principles were defined as:

- *Inclusivity* is about involvement of a *diverse* range of people, and *equal* opportunities for people to become involved irrespective of their social backgrounds and abilities.
- *Partnership* relates to researchers and involved public members showing *respect* for each other’s contributions and roles, and working together in teams.
- *Purposeful* involvement has clarity on why members of the public are involved in research and this is communicated to everyone involved. There is a commitment to involvement.
- *Transparency* is about open and honest communication between researchers and public advisors, and clarity on why things are done in certain ways.
- *Value different kinds of knowledge* recognises that public advisers have complementary expertise to researchers' technical knowledge.

**Fig. 2:**
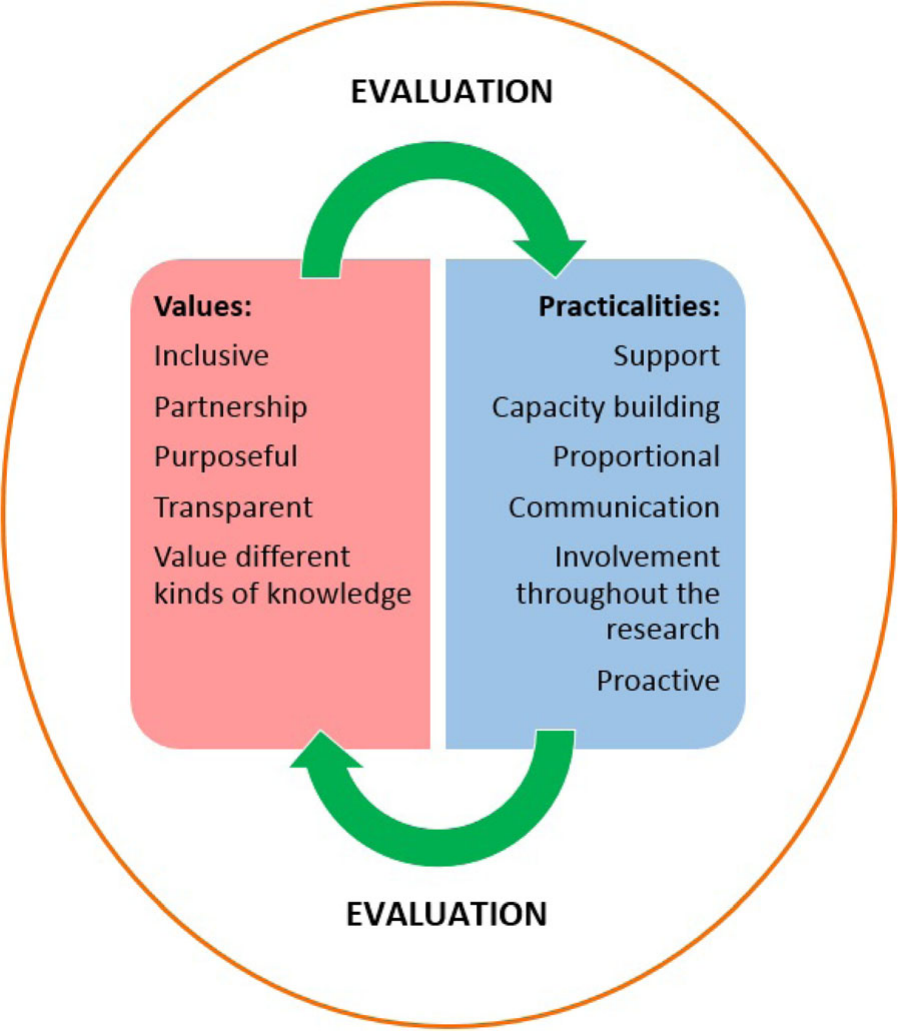
Relationship between involvement values and practicalities

The six practicalities were defined as:

- *Support* to public advisers means a budget for reimbursing travel and time, and dedicated staff who attends to individual needs before, during and after meetings (for example; vision aids, disability access), and who advocate for involvement within the research institution.
- *Capacity building* relates to co-learning between public advisors and researchers, and training for both groups.
- *Proportional* involvement is tailored to the needs of the research and public advisers, and pragmatic decisions are made to balance contradicting demands and limited resources.
- *Communication* is closely linked to the principle of support, and needs to be responsive and *proactive*. Public advisers need to be updated on how the research progresses, and the communication mode should suit the needs of public advisers.
- *Involvement throughout the research* means that there should be opportunities to be involved as a patient, carer or public adviser in any stage of the research. The depth of involvement is likely to vary by each stage, but this practicality enables the (value principle) of “valuing different kinds of knowledge” (Fig. 2). Involvement throughout the research is practically enabled by having infrastructure, leadership and a governance framework for public involvement.
- *Evaluation* means identifying good practice, through communication, research and learning from each other.

The involvement values and practice principles are brought together in Fig. 2 above. The green arrows illustrate the circular relationship between these principles. When planning involvement, it can be helpful to start with the values and consider the practical principles that flow from these. Evaluation is an ongoing need in involvement and the overarching purpose of this article is to report one way of addressing this principle.

### Research question 2: how do these principles for good involvement, identified in the literature, compare with the views of members of our three involvement groups?

Six, seven and 19 members attended each of the three group-specific co-production workshops. We did not collect demographic characteristics of attendees since this is not common practice for involvement meetings. The primary aim of the co-production workshops was to create a space for critical reflection and discussion within each group; enable open sharing of what helps good involvement within the groups, and what the challenges are. The prioritised statements and words from the groups are shown in Table 3 below.

**Table 3:** Group statements and words prioritised in the workshops

Family Faculty/ PenCRU	PenPIG/PenCLAHRC	Adviser Forum/CLAHRC NWC
How is this group best described in terms of what it does, and how it is set up?		
Flexible membership – Family Faculty members can dip in and out of involvement in a way that fits flexibly around their lives, commitments and priorities Influencing research at all stages Partnership with researchers and parent carers, with the Family Involvement Coordinator role at the heart Diverse experiences of parents of children with different conditions and ages A safe, non-judgemental environment which allows researchers to tap into the wealth of information and knowledge stored in parent’s heads	*What PenPIG does:* Public voices: not just one opinion, diverse experiences, perspectives. Hands on/Activities: helping with research, PenCLAHRC management, students, reviewing proposals, academic papers. Interpretation: layman’s language, critical view. *How is it set up:* Supported by PPI team with some self-governance. Well financed. Training Members have different life experiences and varying backgrounds.	Themes identified in discussions are (no order of importance): Governance Sharing personal experience/research projects Training Collaborate/knowledge exchange Dissemination Resources the PRP have developed Values and reach
What makes this group work?		
Family Involvement Coordinator creates a safe space for work to happen Involvement can be fun – meetings are not too serious, and there is fun and laughter. Family Fun Day as a way to say thank you to families Enthusiasm The fact that “I’m not just a parent carer” or “I’m not just a researcher” Lunch is provided at meetings	Teamwork: working together, using the diversity of knowledge and experience within the group Skills (attributes) of PenPIG: goodwill, volunteerism and the ability to give their opinion Research projects: important research put forward for involvement. Support from PenCLAHRC for PPI: refreshments, expenses and participation payments. Staff: mutual respect between staff/researchers and PenPIG, a feeling of being valued. Passion of PenPIG: wanting to change things for the future.	In order of importance: Valuing each other’s experiences, opinions, values Good leadership from the PRP staff. Good facilitator, willing to listen. Good leadership, keeps group focused on tasks relevant to the group. Working as a team. Recognising our strengths and weaknesses Sharing personal experiences
What are the challenges facing this group, or being a member of this group?		
Not hearing how involvement has made a difference Group dynamics can be tricky with dominant personalities Lack of confidence – speaking up in a group or realising you can contribute valuable input Meetings can run over – vague meeting finishing times Getting new members to join the Family Faculty – where and how to integrate without intimidating Not always clear what is expected from Family Faculty at meetings – sending meeting agenda in advance is useful	Potentially/occasionally poor etiquette: lack of respect, focus, or attention to group rules could threaten the valued group dynamic. Relationship of members: differing opinions, stale membership and strong personalities. Practicalities of attending meetings: geographical spread, lengthy travel times, an individual’s health or the timing of a meeting. Finance (sustainability): Funding for the future. Lack of feedback: rushed meetings and lack of feedback afterwards.	In order of importance: Uncertainty about the future? CLAHRC continuing? Sustainability for existing members in future work Staying involved in project opportunities How can we demonstrate public involvement contributing to a reduction in inequalities in health Staying united. Change in dynamics with new ways of working

These statements speak clearly to three of the five value principles identified in the literature: ‘value different kinds of knowledge’ (*not ‘just’ a parent carer*), ‘partnership’ (*teamwork; working together*), and ‘inclusivity’ (*diverse experiences; a safe non-judgemental environment*). A statement on challenges relates to the ‘transparency’ principle (*not hearing how involvement has made a difference; lack of feedback*). In PenPIG the value ‘partnership’ both corresponded to what makes the group work (*mutual respect*) and the challenge (*differing opinions and strong personalities*). Similarly in the Adviser Forum the value principle ‘purposeful’ was implemented through the involvement facilitator (*good leadership, keeps group focused on tasks relevant to the group*) but the same principle was also a challenge (*how can we demonstrate public involvement contributing to a reduction in inequalities in health*).

Group statements address four of the six practice principles from the literature review. The practice principle ‘support’ was valued across the groups, as dedicated university staff to support involvement, financial reimbursements for travel and time, food at meetings, and administrative support. Similarly the principles ‘Involvement throughout the research’ (*helping with research, PenCLAHRC management, students, reviewing proposals, academic papers*; *influencing research at all stages, governance*), ‘capacity building’ (*knowledge exchange, training*) and ‘proportional’ (*flexible membership*). None of the groups’ statements correspond well to the practice principle ‘evaluation’, and challenges were identified in relation to the ‘communication’ principle (*vague meeting finishing times, lack of feedback*).

The workshop highlighted important aspects of public involvement that were not identified in the literature review (*meetings are not too serious, and there is fun and laughter, passion, sharing personal experiences, sustainability, volunteerism*).

### Research question 3: what do these involvement principles look like in practice?

Three descriptive narratives (Supplement 1) were written based on the statements in Table 3. These narratives were coded to the values and practice principles identified in the literature review. This helped us to identify how some principles are implemented in these groups, as described below.

#### Value: support

The details below are blended from the three groups since they enact the value of support in similar ways.

##### Example of support in practice

Each group is supported by a team employed by the university. This team is funded through external grants, and responsible for facilitating meetings and information flow between public advisers and researchers. All three teams have someone in an administrative role and a designated involvement coordinator.

The support provided includes: communication before and after meetings, booking or reimbursing travel and accommodation, explaining items on upcoming meeting agendas, and attending meetings with public advisers.

Before a research meeting the involvement coordinator or administrator will contact people to ask whether anyone has particular needs in regards to accessing the room, diet, or participating in the discussions. If needed they will organise an interpreter or a transcriber to be at the meeting. The coordinator or administrator will always try to see the room in person before booking it, because some rooms marked as having disability access are still cumbersome to use. The teams have a budget for paying public advisors’ time and travel.

Poor health or caring responsibilities can prevent public advisers from attending a meeting and they might want to stop their involvement periodically. If the public adviser prefers, someone from the involvement team can inform the researchers about the changed situation on their behalf.

##### Challenges to ‘support’

All groups identified future funding uncertainties as challenging to providing ‘support’. We hope that our description above has illustrated what good support can look like, and be used as an argument for resourcing involvement support adequately. For example so that the challenge ‘attention to individual needs’, identified in the groups, can be addressed. Considering the time and effort requested of public advisors when they are involved in research, the groups considered adequate and resourced support as essential and necessary for sustained involvement. Providing support also speaks to the principle of partnership and shows a commitment to involvement.

#### Practicality: proportional involvement

The groups rated ‘proportional’ involvement highly. Proportionality might be interpreted as a way of limiting people’s involvement in research, or enabling people to balance their involvement in research with other demands, such as being parent carers, attending hospital appointments or employment. ‘Proportional’ can also mean that the scope of the involvement reflects the size of the research study. Involvement that is proportional includes the principles of ‘pragmatic decision-making’ and ‘diversity across activities’.

We defined ‘pragmatic decision making’ as taking into account the level or rigour, time, resources, and effort required to involve people and to be a public advisor. We cannot always achieve what we aspire to, so we need to be realistic about what is possible. ‘Diversity across activities’ is both about the variety of roles that a member of the public can take on, and activities connected to those roles. There should be options for people to be involved at the level that works for them.

##### Example of pragmatic decision-making in involvement

Members of PenCRU’s Family Faculty can ‘dip in and out’ of research in a way that fits with their personal lives, and without having to justify their varying levels of engagement. Families of children with long-term health conditions live complex lives and fluctuating circumstances dictate their time available. PenCRU Family Faculty meetings are held during school hours since that suits more members who are parent carers. Not all members can attend meetings at these times and those unable to attend are therefore given the option of inputting by email or phone. During meetings, members of the Family Faculty often want to be available should their children’s school get in contact in an emergency, so parents are can keep their phones on at meetings and leave the room if taking a call.

##### Example of diversity across involvement activities

Members of PenPIG are involved in research as lay reviewers, research advisors, co-authors, presenters, assistant teachers, co-applicants, members of advisory groups and representatives in the governance structure of the research programme (PenCLAHRC). Within each of these roles there will be a range of activities: responding to emails, attending meetings and conferences, preparing presentations, reading research applications and preparing for meetings. An example of ‘diversity of activities’ is illustrated by the involvement history of HB, co-author to this article. When she first jointed PenPIG she would only attend workshops to inform research ideas. As she gained confidence, she started to review plain English summaries and full research applications. After some time she volunteered to present at conferences, and later on representing PenCLAHRC at national meetings. She has assisted a member of the PenCRU Family Faculty to deliver training to researchers and other public advisers. With this article she has been involved in evaluation and writing for publication. Being involved in research in a variety of ways has enabled HB to develop her skills, and because of this she has decided to start studying part-time, after years of working and parenting.

##### Challenges to ‘proportional involvement’

Our evaluation did not identify specific challenges to this principle, but the groups’ ability to implement this principle depends on to their financial resource (in turn related to the principle of support).

#### Value: partnership

Partnership and respect is about public advisers feeling valued, respected and seeing that their involvement makes a difference to research.

##### Example of partnership and respect

In NWC CLAHRC community members from the Adviser Forum attend management team meetings, steering board meetings and are members of the committee that approves research funding. Membership on these teams signals to public advisers that they are important partners in the NWC CLAHRC. To equip the advisers for these meetings, the public involvement coordinator meets with advisers to discuss the meeting papers, debate the content and agree a group ‘stance’ on matters of importance to the Adviser Forum. The coordinator also attends meetings with the public advisors and assists them in reporting back to the Adviser Forum. A mentoring programme was established for advisers with a governance role. Advisers were matched with a senior member of NWC CLAHRC who supported them in carrying out their governance role.

##### Challenges to ‘partnership’

One source of difficulty in regards to the ‘partnership’ principle is the inequality between a resourceful university setting and public advisers who are involved in a personal capacity. This can occasionally play out at meetings if a group member challenges the research in passionate ways that are perceived as anger, or displays discomfort in participating in the university space.

#### Value: valuing different kinds of knowledge

This principle speaks to the inequality of knowledge status when different kinds of experts meet. In good involvement everyone’s knowledge, perspectives and experience is valued and judged on their relevance to the research rather than who contributes.

##### Example of how different kinds of knowledge are valued

PenCRU research staff and members of the Family Faculty have co-developed ground rules for meetings which state that nobody is “just a researcher or parent/carer or professional”. This is further demonstrated by the inclusion of Family Faculty members as co-applicants on grant applications, co-investigators on funded projects and equal members of study management groups.

For example, the NIHR-funded Healthy Parent Carers project was co-produced by researchers and members of the Family Faculty, and shows how knowledge from different groups is complementary. The idea for this study was conceived by parent carers at a meeting in 2014. Following this meeting, researchers worked with parent advisors to design and evaluate an intervention to improve parent carers’ health and wellbeing. Parent carers have been involved in co-designing the intervention content and delivery methods, testing programme components, interpreting and disseminating the findings of the initial study, planning the next steps of the research, and recommending ways to respond to funding application feedback. In this study, which successfully gained competitive research funding, the research project team works with parent carers at regular working group meetings, and two parent carers are co-investigators on the study.

##### Challenges to ‘valuing different kinds of knowledge’

It has sometimes been challenging to integrate new members alongside more experienced members at working group meetings. The mixed parent expertise is valued but existing members of the group (researchers, parents and facilitator) also want to avoid repeating discussions and decisions that have already taken place previously. To address this, new members will usually be invited to new rather than existing projects. Pre-meetings to inform new members of where the study is at can also help, or having an introduction period when new members observe meetings before becoming active members.

#### Involvement principles that are difficult to implement

Two value principles were identified as more difficult to implement: transparency and purposeful. The groups said that the purpose for them being involved is not always clarified, nor what is expected of public advisers at meetings. This relates to the potential inequalities between university-funded researchers and volunteering public advisers described earlier, which can challenge transparency of relations. Having clear role descriptions and frank discussions about expectations at the start of the research will help with this. It is best if roles and expectations are written down, so that public advisers and researchers can refer to them throughout the collaboration. The process of writing it down can in itself clarify purpose and expectations. Another recurring challenge is feedback to public advisers on what happened as a result of their involvement, speaking also to the practice principle ‘communication’. To address this, one of our groups has adopted a published feedback questionnaire [21].

Our evaluation found that sometimes a principle can be partially implemented. One example is ‘inclusivity’. PenPIG membership was initially set to a maximum of two years. Approaching the second anniversary of the group some members expressed concerns about having to leave a thriving and positive partnership. The two-year extension was subsequently removed. From the researchers’ perspective it is helpful to have members with long experience of involvement in research, and they alternate between involving new people and PenPIG members in research. But there is a waiting list for PenPIG which means that some people are being excluded from the group and the more in-depth involvement this offers in regards to training and participation in governance. To address this issue, the involvement facilitators and group members will develop a joint strategy on how the group will expand without losing existing members. In addition, many involvement opportunities are advertised to everyone on the team’s mailing list. It is exactly as these kinds of challenges are identified that the investment in support helps ensure collaborative solutions to improving implementation of involvement principles.

#### Good involvement practices not reflected in the research-based involvement principles

Members of all the groups emphasised passion, wanting to change things for the future, and enthusiasm as important to ‘good’ involvement. Informal and welcoming meeting spaces, and an element of ‘fun’ were also identified as positive aspects of involvement. This included the importance of members coming together and sharing experiences as patients and carers, as well as valuing each other’s experiences and opinions. Having space for this within the groups were seen as indicators of ‘good’ involvement.

## Discussion

We have reported on the process and outcomes from a self-reflective co-produced evaluation of the implementation of values and practice principles for involvement in research. This evaluation was prompted by queries from colleagues elsewhere asking for details of how we ‘do’ involvement in practice and an opportunity that arose through the learning exchange to compare across the groups. We sought to explore ways of critiquing our own experiences and assumptions about our practice of public involvement. We aimed to do this in a transparent way, without funding, which might be the same context for other groups wishing to evaluate their practice.

In our groups this evaluation has helped public advisers and researchers to improve our involvement practices. This process has highlighted to us that there are different ways of doing ‘good’ involvement, although some practices are consistent across these three groups, particularly in relation to ‘support’. Some of the identified challenges have been relatively easy to address, for example more clarity on meeting times and providing clearer feedback on people’s impact on research. Other challenges are harder to tackle, such as sustained funding. The process of synthesising the literature into principles, and using these to code our own practices, enabled discussions across the groups and individuals about how we understand involvement, and how we translate our understanding into practice. Some of the impact from this is subtle, such as a heightened awareness in all of us of certain concerns in the groups, or heightened understanding of other people’s priorities.

People are likely to have different aims and aspirations for being part of a collaboration, which can make evaluation difficult and it’s important to acknowledge that context is a key driver for what good may look like [12]. Aspects of involvement that can be researched include: how meetings are facilitated, what people do, the outputs resulting from the involvement, characteristics of the relationships formed and impact from involvement [22]. Impact, in turn, has been considered in relation to nine areas: the research agenda, the research design and delivery, research ethics, public advisors, researchers, research participants, the wider community, and the implementation or change resulting from the research in which people were involved [23]. The evidence-base for impact is growing [24–28] and is of importance to researchers and public advisers alike [23, 29, 30].

We have demonstrated one way of engaging public advisors in reflecting critically about involvement implementation and practice. Our experiences show that involving public advisors collectively in self-evaluation can bring to light important aspects of involvement that have not been emphasised in the academic literature on principles, particularly the benefits of involvement experienced by public members, and the importance of feedback about their input on research. This chimes with other evaluations [21, 29]. The public advisers in our groups also expressed concern around long-term sustainability of their groups since they are all dependent on funding grants.

Researchers seeking tips for involving public advisers might be more interested in practicalities than adherence to principles. We advocate that aspiring and striving to meet the principles outlined in our literature review is essential to achieving impact, and also essential to ensuring that the involvement experience is positive and sustainable. Our evaluation highlighted ‘fun’ and enjoyment, emotion and sharing lived experiences as essential to making involvement a meaningful and worthwhile activity for public advisers, and while this repeats findings from other evaluations, these elements are not present in the standard list of principles [29, 31, 32].

We found eleven recently published reports on principles for involvement. This suggests that there is saturation in the literature in regards to generating principles and understanding involvement values, and we now need evaluations and analysis on how to implement these. Such evaluations can also help us to understand mechanisms of involvement and how these interact with impact.

Our evaluation further suggests that research could focus on how to capitalise on the enthusiasm of public advisors and welcoming strong personalities without silencing others. Public involvement happens in several spheres, not just academia, and extending our gaze to other fields could also improve public involvement in research.

### Limitations

We had to be pragmatic in our approach to evaluation. We carried out a structured but limited review of the literature. Other groups might opt to only work with one set of published standards [8]. We also prioritised conversations and discussions over collecting data on particular involvement outputs. Our interest was in the process of involvement, and in how to open up conversations within and across the three groups on potential improvements. Our evaluation is therefore particular to our own context and based on individual views within the groups.

The people invited to the workshops were all active members of the groups. Since the workshops were not designed to collect data from people we did not ask them their ages or ethnicities. This limits the generalisation of our work, but our aim was to showcase a co-production approach to self-evaluation rather than generating generalizable findings.

Some perspectives on involvement are no doubt missing in our work. For example, one author of this article (HB) recently experienced a sense of loss when she had to reduce her involvement activity due to returning to full-time work. This need for support at the end of involvement was not raised at any of the workshops, possibly because the people attending were those still able to be involved in research. The importance of ending involvement collaborations well has been discussed elsewhere (https://evidsynthteam.wordpress.com/2019/05/07/collaboration-in-research-reflections-on-reciprocity/).

## Conclusion

There is a growing body of evaluations of public involvement on impact [24, 26–28] and on barriers and levers to impact and partnerships [29, 33–35]. Our co-produced self-reflective evaluation focused on implementation of involvement principles. We did this by assessing, with public advisers, how the involvement practices and priorities of our three groups fit with the five values and six practice principles identified in our literature review. We have found the process described here helpful in opening up constructive conversations within the involvement groups about challenges experienced. Further information on the details of this process are available from the authors who are keen to work to the principle of ‘sharing involvement experiences’ within the practice principle of ‘evaluation’.

## Supplementary information

**Additional file 1.** Group narratives.

## Data Availability

The literature review data generated from this study are available on request from the corresponding author. The article is not drawing on data that is suitable to a data depository. Further information on how we facilitated the workshop meetings is available from the corresponding author.

## Abbreviations

NIHR: National Institute for Health Research
NWC CLAHRC: Collaboration for Leadership in Applied Health Research and Care North West Coast
PenCLAHRC: Collaboration for Leadership in Applied Health Research and Care South West Peninsula
PenCRU: Peninsula Childhood Disability Research Unit
PenPIG: Peninsula Public Involvement Group
