# Supplementary material for: Public involvement in health research: what does ‘good’ look like in practice?
Source: Res Involv Engagem. 2020 Mar 31;6:11. doi: 10.1186/s40900-020-0183-x (PMC7110674; doi:10.1186/s40900-020-0183-x)
Supplement: Supplementary file 1 — Additional file 1. Group narratives. [file 40900_2020_183_MOESM1_ESM.docx]

**Public Reference Panel: CLAHRC NWC**

**STRUCTURE**

The PRP is made up of members of the public who are actively involved in the research and implementation activities of CLAHRC NWC (public advisers), usually recruited to work for a particular research theme project. Public advisers are involved in a variety of different roles, including, but not limited to: sharing their experiences of having a particular health condition; living in a certain neighbourhood; or getting involved in parts of the research process (such as looking at the evidence that teams have collected and trying to make sense of it). Some public advisers may also be involved in pieces of work that are led by the public advisers and supported by the academic and health/social care professionals. All public advisers, once they have a formal role in CLAHRC NWC, become automatic members of the public reference panel.

The public reference panel is a group that gives a collective voice to public advisers involved in CLAHRC NWC. Being a part of the collective voice gives public advisers an opportunity to shape the way things operate in CLAHRC NWC and a way of getting their opinions across and PPIE on the agenda of senior governance and strategic groups. The public reference panel is an open meeting for all advisers, it meets bi-monthly, and advisers can drop in as a one-off or attend every session. Because this is the meeting where a lot of advisers get together at once, we also put on some formal training sessions after the main meeting to support public advisers to carry out their role. Training sessions have been on various topics, including effective influencing skills, public speaking and health inequalities. Public advisers take a lead in identifying the training that they need to carry out their roles, they are also responsible for helping to identify what training the paid members of the team might need to help them to involve and engage the public. An Adviser Experience and Training sub-group of the public reference panel take a lead on identifying this training (this has been done by carrying out a needs analysis with staff and partners and for advisers, through discussions in public reference panel meetings).

The public reference panel has a broad range of responsibilities which are linked to the CLAHRC public engagement policy. Advisers can be involved in a variety of ways, some advisers are more active than others and take on more tasks and responsibilities. Some advisers also join sub-groups which have responsibility for leading work in a certain area (which meet bi-monthly). Meetings are rotated around the NWC region with new venues being tried often as some can be unpopular. Meetings are frequently held at one of the University buildings but only if this is the most convenient- hotels and other meting spaces are also often used to make meeting as convenient as possible for advisers.

Within the public reference panel smaller sub-groups have been set up to carry out specific pieces of work within that theme area and to have responsibility for certain areas. The three current sub-group topics are ‘Governance’, ‘Adviser Experience and Training’ and ‘Media, Events and Communication’ though more sub-groups can be set up as needs arise. Advisers within the Governance sub-group have formal positions on the Management team, Steering Board and Sub-committee. Advisers in the Adviser Experience and Training sub-group take a lead on training and those in the Media, Events and Communication sub-group will be a primary point of contact for the internal evaluation.

Specialist training and support is sought and delivered for advisers carrying out particular roles, for example public advisers who attended governance meetings were given the chance to be part of a mentor scheme with partner and University staff to help them in their role. Another example is when public advisers attend the Sub-committee, they have a training session on reviewing research proposals because this group approves the research projects for funding. We carry out training as we want public advisers to feel confident making contributions and comments about the work they’re involved in.

**Diagram of CLAHRC NWC and Public Advisers within it**


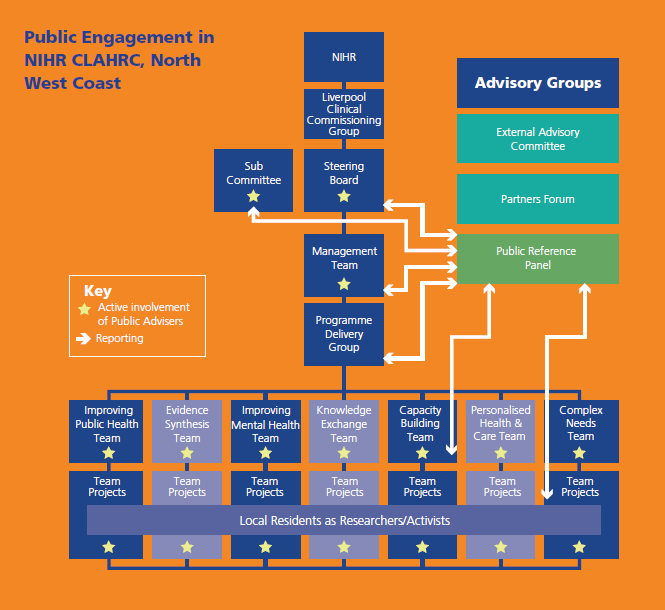


‘The PRP to me is the central station where we all came together. We worked out our destination and planned our journey for how we will build public engagement within CLAHRC (NWC)’. The PRP commented on the vision for involvement within CLAHRC NWC being very important to their success. This PE policy was developed in consultation with two public advisers, before the main PRP was in place. It sets out the principles for involvement (for all staff) as well as the key responsibilities of the PRP group (strategic oversight of the PE policy with specific responsibility for training for example). More formal infrastructure arrangements such as advisers having formal positions on the governance groups of the Steering Board, Management team and Sub-committee were also set out within this document and this supported the advisers to become well integrated into the CLAHRC NWC once they joined and the PRP was formed.

The structures have been created to specifically reflect and embed the underlying principles of CLAHRC in the work that we do: i.e. that Public expertise is ‘recognised as an essential complement to professional expertise and research evidence’. Structures are enabling the public to contribute fully.

**PROCESS**

Public advisers are usually recruited to research or implementation activities linked to one of the overarching CLAHRC research theme areas. Advisers can choose to work in this initial role only or to join the public reference panel. The PRP developed induction materials including a welcome pack, online short video clips and an induction checklist (for research team members) to ensure that all advisers joining CLAHRC are well informed about their role. It’s the responsibility of the person engaging the adviser in the initial research/implementation project to use these materials to induct the adviser. Additional induction sessions are held before each open panel meeting to give advisers new to the panel a better understanding of the group and the roles they can play in it. When attending their first meeting, new advisers are given a ‘buddy’ for this meeting- a member of the group to support them in the meeting and over lunch.

After panel meetings, training is put on to support advisers in their role- attendance is optional though those attending a training topic to support them to carry out a specific role they have been asked to do within CLAHRC will be offered payment. Once attending the panel, advisers can stay a part of the large open (bi-monthly) group or join one of the working groups (also meeting bi-monthly on the alternate month). These working groups are needs-based and are set up on topics that help to deliver the public engagement policy. The sub-groups are limited in size to around 8 members and membership is reviewed each year to allow for turn-over of membership which is managed through the main PRP meetings. Advisers are ‘coached’ on their role as a public adviser through the PRP by the PE facilitator (a full time member of staff) and long-standing PRP members act as .

Training is delivered on a number of levels. At research project level, teams will have identified in their bid if specific training for advisers might be needed (for example carrying out peer interviews). General adviser training is put on at PRP meetings on topics that are of relevance to many advisers (a needs audit is carried out periodically to set the topics). On occasion, an adviser or the team working with the adviser may find that either the role of the adviser has developed further than planned (for example becoming involved in carrying out a systematic review) and in these cases local training will be put on to support the adviser to carry out the role. For research teams, partners and other members of staff, a needs assessment is carried out periodically to find out the needs and gaps in knowledge and understanding in PPIE. Training will be delivered to meet these needs. In all cases there may be a mix of internal and external training providers, depending if the expertise is already available in the collaboration.

Within the PRP the advisers are actively involved in the delivery of the PE policy. On occasion this might mean carrying out a specific piece of work (for example taking forward the finishing off of a policy that has been discussed at a large PRP group or delivering training for PHD students about being a public adviser and what’s needed from the teams advisers work with. When these opportunities to be further involved in our work come up, the PRP agreed that they would prefer the PE facilitator to allocate the opportunities. Advisers just need to express an interest in the opportunity and the PE Facilitator tries to share our opportunities across the group, balancing opportunities for development and matching skills to the opportunity. Usually two advisers are selected for opportunities as it might provide an opportunity for one adviser to develop their skills and another may feel confident with the task.

The PRP gives advisers an authentic opportunity to influence the way the CLAHRC NWC collaboration works. It is a group facilitated by the PE Facilitator and democratically run which means that advisers can see changes put in place as a result of their involvement. Examples of the reach and influence that advisers have had on the collaboration include the PRP carrying out a piece of work to develop metrics around the CLAHRC NWC principles for engagement. The group looked at each principle, developed a corresponding entry on the risk register (adopted by the Steering Board) and offered recommendations for ways of working (or minimum standards). By visualising what living our principles would look like, the group successfully instigated a number of changes via the Steering Board. Research projects now report additional information in their quarterly reports, governance groups agreed to adopt ‘action logs’ relating to public involvement, and detailed data linking engagement to theme areas is now reported at the Steering Board. This piece of work helped PRP members to trust in the collective power of the group.

**OUTCOMES**

By taking part and being offered a wide range of research, implementation, formal governance and administration opportunities, advisers report a feeling of appreciating the personal development that being involved in the CLAHRC NWC has given them. Advisers have commented on an increase in confidence, self-worth, self-esteem and personal value as a result of being a part in the work. Feeling trusted and valued are also outcomes of involvement in CLAHRC NWC that were often reported by the PRP group.

When discussing the challenges, the PRP the group tended to focus on the risk to continuity from fixed term funding and commented on the challenges of uncertainty within an organisation. Alongside the external challenges there were concerns about the changes currently underway within the PRP. After a period of over 2 years as one group, the shift to operate in a sub-group model, and new members joining the group has created nervousness, as with any change. The PRP, though supportive of the change were keen not to lose the elements of the group that helped make it such a success. The new model still has an open PRP group meeting (for any adviser involved in CLAHRC NWC), though rather than monthly, this now happens on a bi-monthly basis. Training is still delivered on the afternoon on this meeting wherever possible. On the alternative months, this is when the Media events and communications as well as the Adviser experience and training subgroups meet. These two subgroups have their own agenda linked to strategic areas of CLAHRC NWC and carry out pieces of work linked to these theme areas. The third subgroup (Governance) also meet between the open meetings but this group has ‘pre-meets’ which take place before the governance meetings (management team, steering board and sub-committee) to help prepare advisers attending these meetings and to agree what will be presented at the meeting. At the open meetings these three subgroups have time on the agenda to talk to the open panel meeting about the key areas of work within their subgroup and use the meeting as an opportunity to get feedback from the group and ensure that the panel consensus/opinions are fed in to the work of the subgroups and any comments made at governance meetings (so individual advisers and pieces of work are representing the whole adviser group. Adviser numbers at the monthly open meetings had been steadily growing and decision making in a PRP with numbers of 16 was just manageable. As numbers would only increase, the decision was made to refresh the model. The principle of all advisers becoming automatic members of the PRP is an important one and so was protected by developing a new way of working which would allow a larger group to come to the open PRP metins as well as have decision making take place and pieces of work completed in smaller, more manageable groups. This new model also allows advisers to specialise in a particular area. Adviser numbers are over 100 across CLAHRC NWC and open meetings attendee numbers flux and can attract up to around 30 advisers, though no two are the same.

# Patient and Public involvement in practice: Family Faculty **(PenCRU)** Model

## **Structure**

The Family Faculty (FF) is an integral part of a university-based childhood disability research unit known as PenCRU (The Peninsula Cerebra Research Unit for Childhood Disability).

The unit was established in 2009 with the remit to bring together researchers, healthcare professionals and families/carers of disabled children to work in a partnership to ensure that research relating to childhood neuro-disability is meaningful, relevant and accessible to those whose lives are affected by childhood disability.

People become members of the Family Faculty by completing the online form or providing their details to PenCRU. There are currently around 350 members who have a diverse range of experience and expertise in supporting children with a range of conditions, such as autism and cerebral palsy, and of different ages. Members have no obligation to be involved in the work of the unit, and can limit their involvement to receiving information on PenCRU’s activities via emails and newsletters. Members dip in and out of active involvement in a way that fits flexibly around their lives, commitments and priorities. Typically there are around 30-40 more active members that either participate in meetings or contribute remotely. The more active parents vary depending on the topics of projects and their own circumstances. Meetings are usually held during school hours in term time as that suits the preference of more members. A member can contribute by email/phone in advance if they are unable to attend the meeting. Although meetings are scheduled taking into consideration other commitments, it’s inevitable that not all members can attend meetings.

Meeting and other opportunities are advertised to the Family Faculty list by email. Some projects focus on generic topics on which most parent carers can contribute their experience; other projects focus on specific conditions where parent carers generally self-select to volunteer because their child has that condition. Family involvement includes remote work for example reading draft articles or responding to surveys, and also attending workshops, training, conferences and meeting(s) hosted by PenCRU staff related to a particular research project working group. FF also review the PenCRU research evidence summaries, to ensure the language used is clear.

Attendance at meetings is supported by reimbursement of reasonable travel and parking expenses. The cost of associated childcare is also reclaimable, however due to the university’s strict policy, which requires that the provider must be Ofsted registered, many arrangements that members make for their children are exempt from this offer. This is because many disabled children need to be left in the care of a person they know, and who knows their needs, such as care staff from their schools. These people are well qualified but offer care on a self-employed basis and are not required to be Ofsted registered in that context.

Acknowledgment payments are made to Family Faculty members for their contributions using our payment policy which is on our website. The funding for this comes through NIHR. Travel expenses can also be reimbursed, which we fund via our core support from the charity Cerebra. Payment is not designed to be a financial incentive to attend but to show value for the person’s expertise and time that they contribute freely. This money is counted as income for the members for tax and benefit purposes; some parent carers elect for us to direct their payment to recognised community organisation or charity.

Cerebra funding also supports some of PenCRU staff. A long standing senior researcher leads the team of a number of researcher positions, key administration support as well as a dedicated Family Involvement Coordinator. The role of Family Involvement Coordinator is central to the work of the unit, acting as the primary liaison with families with disabled children, and coordinating and supporting their meaningful involvement in all our activities. This role is highly valued and considered essential by FF members. The Family Involvement Coordinator is the main point of contact between families and researchers, and support effective relationships to foster partnership. The role was purposely designed not to be a researcher who had to focus on academic career, projects, papers and grant applications, but be more focused on ensuring public involvement was well planned and delivered appropriately.

PenCRU staff recruitment process includes at least one but usually two FF members at the interview stage. This aims to ensure PenCRU staff are empathetic, compassionate, warm and approachable personalities who are likely to be suited to working in partnership with the Family Faculty.

PenCRU have office space in the medical school of Exeter University’s with access to bookable meeting rooms on the campus. Due to logistical expediency, most meetings involving the FF occur in this setting which means that the active membership is concentrated within the commutable zone of the city in the rural county of Devon, in the south west of the UK. External community venues are feasible but more expensive.

The campus is located near to the city centre, close to a range public transport links. However, the rural nature of the area and the need for members to fulfil their caring responsibilities compels most members to arrive by car. Car sharing is practiced and car-parking is available onsite. It is free of charge to FF members via the university visitor parking permit system. Parking permits are sent to members before a meeting. However, space is limited and a valid permit does not guarantee a space to park. This is a considerable source of frustration of members who frequently have to search for on street parking nearby or go to one of the city centre car parks. As parents, members of the Family Faculty typically want to be able to be available should their children’s school get in contact.

The building is a modern and new, set around a tranquil green. It has accessible entrances, lifts and toilet facilities. On arrival to meetings members have access to refreshments, tea/coffee and biscuits, and afterwards lunch is provided. This is greatly appreciated by members of the Family Faculty. The team do not use the universities official catering team formally claiming it is because an independent one is cheaper (which it is) but less formally explaining the preference is based on the quality and choice available.

## **Process**

Involvement activities are underpinned by the expectation that they will be characterised by meaningful and respectful partnership working. In the ground rules (handbook and recited at meetings) participants are challenged to perceive themselves as more than ‘just a’ researcher/parent/carer/professional all are valuable contributors in a non-judgemental dialogue.

There is no written role description. Success relies on putting aside personal agendas, to listen with an open mind, to learn from each other and to work towards achieving the best outcomes for the research task at hand. This requires much enthusiasm and effort on the part of researchers and FF members alike.

A significant barrier to achieving this is biggest when FF members are new. In this situation there is the need for PenCRU to explain what being involved in research means. The constant assertions that ‘there is never a stupid question’ and that ‘no experience is needed at the start’ are useful to assist a new FF member develop the confidence they need in knowing they have something useful to contribute. Although there is a process for the Family Involvement Coordinator to induct and welcome new members when they join and attend their first meeting, and we have discussed a buddy-system, the group can take time to accommodate new members. Also it can take new members time to understand and feel empowered to contribute effectively. There can be a real tension between maintaining that flexible approach to work to orientate new members and the group getting on with the work at hand.

The orientation process is aided by reference to the research cycle* (share this) and that research strands are organised into project working groups to offer continuity. Opportunities to get involved with specific projects are advertised by email and members of the Family Faculty volunteer to constitute a working group that is established for that project. Members are invited to join any working group of interest to them as long as it makes sense for the stage and focus of the project. This is not an easy task when there has been a gap between meetings, while research work has been occurring, and FF members have understandably forgotten the thread and may feel they cannot make a useful contribution.

Many established practices have evolved over time, and they are understood to have benefited from the ideas and suggestions of lots of people; staff/parents, past/present. It is considered important to embrace learning from what doesn’t work and considerable effort is used to be responsive to issues as they arise.

Healthy involvement is now strengthened by a FF handbook. This sets out, amongst other things, a code of conduct. This handbook was produced in partnership between PenCRU and the FF, and clearly sets out expected reciprocal behaviours that need to be observed to make involvement work. This mutual understanding of expectations creates a friendly environment that values FF members as individuals, and helps them feel safe and included.

PenCRU staff work hard on pre-meeting planning, often responding to various practical issues that come up. Meeting details and an agenda are sent to FF members in advance, where possible. However, for PenCRU to be able to address specific expectations, such as dietary requirements, these need to be communicated to the team in a two-way process.

Meetings are preceded by a half hour scheduled catch up time. The aim here is to support flexible arrival times, build rapport between attendees, provide a warm welcome and facilitate a space for people to offload challenging personal situation they have/are facing so it is not discounted but it does not significantly encroach on the formal work.

Transition to the focus of the meeting is assisted by the consistent use of a round of introductions at the start. This is now combined with an ice breaker exercise, which serves to set a relaxed tone and build a sense of commonality.

Management of interactions, when working through the agenda activities, requires self-awareness by FF members as well as a skilled chairperson. The chairperson is usually the senior researcher running the group. The chairperson can redirect the focus of a discussion, when it is invariably going off topic, with appropriate sensitivity when the underlying cause is that an emotionally sensitive trigger has been activated. As a group, patience compassion and acceptance needs to be employed in these situations.

Assertive reference to the code of conduct is needed when the shared conversation space becomes controlled by dominant personalities or other interpersonal clashes. Diversions into someone’s personal agenda requires some tolerance when it is associated with their acute disempowerment of managing a complex disability or caring role. Here it is best to offer another time to unpack that (for example over the lunch).

The PenCRU team now have a number of FF members they refer to as ‘more experienced members’. These ‘more experienced’ members are recognised by the time they have been involved and their capacity they demonstrate to internalise the skills and knowledge needed to be an effective team contributor. Sustaining their continuing involvement is important for the unit as a whole as they can embody the skills new members need to develop. PenCRU has over time looked to offer the ‘more experienced members’ more complex, challenging and rewarding activities such as presenting at conferences. It can be problematic if any other members not invited to an opportunity feel excluded. We have discussed role profiles for these experienced members as ambassadors but no formal process has been established for selection or role description.

Many FF members do not get involved until a research topic that is relevant to them and their experience comes up. Others feel that there are too few relevant opportunities for them. However, PenCRU have noticed a small dropout rate, potentially signifying that these FF members still want to be included in the mailing list to be informed of research involvement opportunities.

## Outcomes

Many of these are personal and intangible. Those deemed most significant include:

- Contributing to making research relevant and robust.
- Making the PenCRU research evidence summaries as accessible for other families, who are not FF members, to use in their treatment decision making process.
- Being able to be develop more creative involvement activities that embed PPI into the research cycle.
- Genuine and trusting rapport with researchers and FF members.
- The development of a secure and non-judgmental space for open and frank discussions.
- FF can enhance their communication and discussion skills.
- Inspiring researchers in their work and supporting them to feel more confident in receiving feedback from FF
- Seeing the spirit of involvement be explored and embodied in more creative and satisfying ways.
- Empowerment of all those involved, especially FF members. Improved wellbeing of members who feel valued and respected.
- Building of confidence and self-esteem.
- The functional benefits of having a voice that is listened too.
- Seeing FF members to develop and grow over time to transcend their caring responsibilities.
- Opportunities to develop interpersonal skills and increase a person’s responsiveness to habitual practices, and open mindset at a deep level.
- Ongoing frustrations of not hearing how specific involvement work has made a difference.
- Awareness of enduring communication problems; including the need to be kept up to date with matters.
- An increased awareness in the challenges to include FF members in the governance of PenCRU as a whole.
- Appreciation of how important good involvement is to research. Being aware that not all researchers respect the contribution of patient and public like the PenCRU team.
- Acute awareness that the research process/cycle takes a long time.
- Being at risk of becoming disheartened or despondent with the process.
- The effective representation of PenCRU at conferences and external meetings enhancing the value and esteem of the name of the university, PenCRU as a research team and the cerebra charity.
- Challenging traditional hierarchical power dynamics by engaging deeply in meaningful involvement.
- Awareness of the challenge to maintain the considerable effort needed to do involvement well.
- The risk that people can be hurt at a personal level when they are fully invested and something goes differently to how they expected.
- The knowledge of the privilege involved in involvement
- Being able to turn personal challenges and impairments into an asset.

**Patient and Public Involvement in Practice: PenPIG Model**

**Structure**

The Peninsula Public Involvement Group (PenPIG) is an advisory group made up of seventeen members of the public, service users and carers who live across the South West Peninsula of England, UK. The original and formal number of the group is 15 but at one point the membership decided to accept two extra members as they felt it would be possible to accommodate them. Fifteen members was originally seen as the maximum possible to include for boardroom style meetings, and to fit with the budget. Originally membership was limited to 2 years, but when the first memberships came to an end the group, in collaboration with the PPI team, agreed that there would not be a time limit. At the beginning, members of the PPI team would actively attend community events to recruit members to PenPIG. In the last two years all membership has been through word of mouth. PenPIG is set up by PenCLAHRC to work with researchers and have representation within the organisation, but the PenCLAHRC PPI team works with additional community members or patients depending on what kind of expertise is needed by any research study. There is no formal communication between PenPIG and other people involved in the same capacity, but people sometimes meet at training events or when an involvement opportunity is open to both groups.

PenPIG members have a wide variety of health and life experiences and come from varying socio-economic backgrounds. Individual members of PenPIG are involved in a wide range of activities including lay reviewing, research prioritisation, co-writing applications for research funding, co-authoring papers and supporting user involvement in on-going research projects. Activities are led by our involvement strategy (<http://clahrc-peninsula.nihr.ac.uk/uploads/attachments/PenCLAHRC%20PPI%20Strategy%20170704.pdf>), by PenCLAHRC needs as they arise, and by initiatives from PenPIG members. The group also has representation on PenCLAHRC’s* Management Board. Initially, two members from PenPIG were selected by the group to attend these meetings. To widen the opportunity, the PPI team decided that outgoing membership and business secretaries will take on the board representation for the forthcoming year. In addition to individual involvement the group meets quarterly rotating the location to cover the wide geographical spread. Meetings are held in any suitable venue that has wheelchair access and parking nearby. All members are invited to all meetings independently of their home county. PenCLAHRC allocates considerable resources to supporting PPI. The financial support enables travel expenses and participations payments to be made as well as providing training events and refreshments at meetings. Training is provided on an ad-hoc basis and responding to needs and interests in PenPIG. Sometimes training needs is discussed at quarterly meetings. Some training is study-specific, some is provided to all members. In the last year PenPIG has informed the development of new introductory training to members of the public. A public involvement team of four research staff and an administrator support both PenPIG and the PPI requirements of individual projects across the region. The research staff facilitate involvement in research, publish about involvement (based on research on involvement or discussion papers) and are co-applicants on research funding bids. Their role as co-applicants span facilitation and academic work: they provide input as researchers onto the study design, they also enable input from patients/carers/public who are not able to come to the meetings. They enable this input by meeting with people outside of the meeting, and informing people of the impact their input has had or not on the research.

PenPIG has some aspects of self-governance. Co-ordinated by their volunteer membership secretary, members have a say in accepting new people to the group. Interested applicants contact the membership secretary with a personal profile. This profile is circulated to all existing members by email, and a decision is made by the group whether to accept the new member. The group’s volunteer business secretary co-ordinate the setting of the agenda for their quarterly meetings. The agenda is not bound to the strategy or CLAHRC priorities, it is entirely set by PenPIG members although the PPI team does suggest items. These meetings are chaired by a PenPIG member (by rotation), and function as an opportunity for members to meet, learn about research, and keep in touch with PenCLAHRC business. Both volunteer roles are time limited to 1 year.

**Process**

PenPIG members are reimbursed for their travel expenses on the day of travel which is seen as essential for their involvement in research as it means they are not left out of pocket. Members give their time freely, however in recognition of their valued contribution, a payment is made.

The selection of members to be involved in research studies and other activities happens on an ad-hoc basis and depends on the nature of the involvement opportunity. Sometimes there is a need for a ‘general’ opinion by a member of the public or there is enough budget to involve anyone willing to give their time, other times a particular type of patient expertise is asked for or there is a limited budget. This means that sometimes people become involved through self-selection, other times they are approached individually due to their patient or carer experience fitting with a research study. Occasionally people are selected randomly, or selection is based on previous involvement activities. This means that sometimes someone is selected because they have missed out on other opportunities, other times someone is selected because they have developed skills related to a particular type of research design.

Meeting researchers face-to-face who listen and show dedication to involvement by acting on PenPIG’s advice enables PenPIG to function and there is a feeling of being valued and mutual respect between staff and members. PenPIG value the research projects put forward, viewing them as important and that, by drawing on the variety of experience within the group, the right member for the right task will be found.

There is currently no systematic way of capturing impact from PenPIG’s involvement in research. However, if the involvement is in a funding application impact will sometimes be described in the application. In the past year the PPI team has collected data on some of their involvement activities which will inform research on impact from PPI.

The sense of working together for a common goal, using the diversity of knowledge and experience within the group, is highly valued by PenPIG. This is supported by members’ typical attributes of goodwill, volunteerism and the ability to give their opinion. The high value PenPIG places on working together is reflected in their consideration of the challenges PenPIG face. Of high concern is the potential that poor etiquette, such as a lack of respect, focus, or attention to group rules could threaten the valued group dynamic. Training on effective participation in meetings has been delivered to PenPIG, and from that training the group produced a set of ground rules which are put up at each meeting.

Lengthy travelling times, due to PenCLAHRC’s geographical spread, combined with other issues such as an individual’s health or the timing of a meeting mean that the practicalities of attending meetings can be challenging. The PPI team administrator organises meetings to ensure they are accessible and teleconferencing is offered but people usually prefer face-to-face meetings.

**Outcomes**

For PenPIG the outcome of their involvement is about providing different opinions and perspectives through the hands-on help they give. There can also be unexpected benefits from involvement such as making new friends, gaining knowledge about research from training and other PenPIG members and understanding research.

PenPIG members are invited to annual reviews with a member of the PPI team where they can raise any issues or development needs they have in their role. Members are encouraged to tell the PPI team about things they do outside the group. However, this was not mentioned by PenPIG in regards to outcomes.

* National Institute for Health Research (NIHR) Collaboration for Leadership in Applied Health Research and Care (CLAHRC) South West Peninsula - or PenCLAHRC. This will probably be defined elsewhere in the complete article.
